# Computational Strategies for the Identification of a Transcriptional Biomarker Panel to Sense Cellular Growth States in *Bacillus subtilis*

**DOI:** 10.3390/s21072436

**Published:** 2021-04-01

**Authors:** Yiming Huang, Wendy Smith, Colin Harwood, Anil Wipat, Jaume Bacardit

**Affiliations:** 1 Interdisciplinary Computing and Complex BioSystems (ICOS) Group, School of Computing, Newcastle University, Newcastle upon Tyne NE1 7RU, UK; Y.Huang61@newcastle.ac.uk (Y.H.); wendy.smith@newcastle.ac.uk (W.S.); 2 Centre for Bacterial Cell Biology, Biosciences Institute, Faculty of Medical Sciences, Newcastle University, Newcastle upon Tyne NE1 7RU, UK; colin.harwood@newcastle.ac.uk

**Keywords:** biomarker identification, transcriptional landscape, machine learning, *Bacillus subtilis*

## Abstract

A goal of the biotechnology industry is to be able to recognise detrimental cellular states that may lead to suboptimal or anomalous growth in a bacterial population. Our current knowledge of how different environmental treatments modulate gene regulation and bring about physiology adaptations is limited, and hence it is difficult to determine the mechanisms that lead to their effects. Patterns of gene expression, revealed using technologies such as microarrays or RNA-seq, can provide useful biomarkers of different gene regulatory states indicative of a bacterium’s physiological status. It is desirable to have only a few key genes as the biomarkers to reduce the costs of determining the transcriptional state by opening the way for methods such as quantitative RT-PCR and amplicon panels. In this paper, we used unsupervised machine learning to construct a transcriptional landscape model from condition-dependent transcriptome data, from which we have identified 10 clusters of samples with differentiated gene expression profiles and linked to different cellular growth states. Using an iterative feature elimination strategy, we identified a minimal panel of 10 biomarker genes that achieved 100% cross-validation accuracy in predicting the cluster assignment. Moreover, we designed and evaluated a variety of data processing strategies to ensure our methods were able to generate meaningful transcriptional landscape models, capturing relevant biological processes. Overall, the computational strategies introduced in this study facilitate the identification of a detailed set of relevant cellular growth states, and how to sense them using a reduced biomarker panel.

## 1. Introduction

The majority of free-living, single-celled bacteria inhabit environments that are constantly changing. As a result, they are subject to periods of stress that are often multifactorial in nature. In response to these environmental changes, bacteria have evolved a range of interacting regulatory circuits that bring about metabolic and physiological adaptations that help to mitigate against the damage resulting from these stresses [1,2,3,4,5,6,7,8]. Failure to adapt results in the organism being outgrown by competitors or cell death. From a biotechnological perspective, where bacteria are grown under a limited set of user-defined conditions, environmental and cellular stresses can lead to suboptimal growth, lower product yields, population heterogeneity and reduced viability [9,10,11]. Understanding how different environmental treatments modulate gene regulation can enable us to identify signals that reflect the onset of cellular stresses and facilitate engineering of strategies that ameliorate their effects. Knowledge of how the global bacterial gene expression profile (i.e., the transcriptome) changes in response to nutrient and environmental stresses is therefore key to recognising when a population of cells encounters conditions that are detrimental to growth and product synthesis.

The transcriptomes of bacteria shift as conditions change. These shifts often correlate with observable phenotypic and metabolic changes [12,13]. A crucial aspect for the biotechnology industry is to be able to monitor transcriptomic profiles, recognise detrimental cellular states and use existing knowledge to optimise industrial production [14,15,16]. However, the determination of the bacterial transcriptional state is still a costly process involving the quantification of thousands of mRNA molecules using experimental techniques such as RNA-seq and microarray. Such costs can be reduced by identifying a set of biomarker genes whose mRNA levels are indicative of a particular cellular state. Consequently, the expression state of the transcriptome in the transcriptional landscape, and the resulting cellular state, can then be estimated by measuring the expression of a few key biomarker genes instead of the whole transcriptome. More cost-effective techniques such as amplicon panels [17,18] and quantitative RT-PCR [19,20] can then be employed.

Ultimately, reporter sensor systems could be engineered to facilitate the real time monitoring of the cellular growth state by monitoring the expression of a representative set of biomarker genes in situ. Once key biomarkers have been identified, sensor circuits can also be engineered to modulate bacterial stresses, such as the load stress induced by the production of a foreign protein [21,22,23,24], through the use of negative feedback control strategies [25,26,27,28].

Previous work in this area has mostly focused on cell responses to specific stress conditions [29,30,31] and the corresponding gene biomarker identification using statistical algorithms [32,33,34,35], lacking the quantitative analysis of global gene regulatory systems under diverse environments. A recent study by Avican et al. [36] analysed the transcriptional dynamics under a pool of stress conditions for various human bacterial pathogens but did not provide assessable biomarkers to monitor the stresses. Kim et al. [37] predicted the cellular states for *Escherichia coli* in unexplored conditions with multi-omics data, but this approach requires relatively high data generation costs. The advent of single-cell RNA sequencing has made extensive gene expression data available for the deployment of machine learning methods to study transcriptional heterogeneities in eukaryotic systems [38,39,40]. For example, clustering and feature selection methods have been widely applied to discover cell subtypes and transcriptional biomarkers for human diseases [41,42,43,44]. However, due to smaller sample size of prokaryotic transcriptomics datasets which are generated with conventional RNA-seq and microarray technologies, and the challenges of obtaining single cell transcriptomic data from bacteria, these data analysis approaches have yet to be fully explored to investigate different cellular states and expression biomarkers for bacterial systems.

To fill this gap, we located a large condition-dependent transcriptomics dataset for Bacillus subtilis [45] and designed a set of computational strategies to capture the cellular transcriptional responses under conditions. We performed the dimension reduction technique UMAP [46] to transform the high-dimensional transcriptomic data into a ‘transcriptional landscape’ representing the transcriptome shifts under different conditions. The graph-based clustering algorithm Leiden [47] revealed 10 clusters of distinct transcriptional states in this landscape, corresponding to different cellular growth states. We applied a rank guided iterative feature elimination (RGIFE) heuristic [48] to discover transcriptional biomarkers that are indicative of the cluster in the landscape. We successfully identified a minimal panel of 10 biomarker genes that together achieved 100% cross-validation accuracy in predicting the cluster assignment and a number of alternative biomarker panels with either larger size or lower prediction power. Finally, we characterised the biology captured by each of these 10 clusters and studied functions of these 10 biomarkers from public databases.

## 2. Materials and Methods

### 2.1. Tiling Array Data and Data Pre-Processing

In this study, we used a publicly available high density tiling array dataset [45] of 269 samples consisting of *B. subtilis* strain BSB1 transcriptomes measured under a wide range of different conditions, including the use of various nutrients, aerobic and anaerobic growth regimes, the development of motility, sporulation and germination, and adaptation to diverse stresses (Supplementary Table S1A, Table 1 from http://genome.jouy.inra.fr/basysbio/bsubtranscriptome/, accessed on 1 March 2021). The broad range of conditions potentially led to diverse transcriptional states, making it an ideal platform to explore the transcriptional landscape of *B. subtilis.* Although the more advanced RNA-seq technology has also generated many relevant transcriptomics datasets, they are usually small in size and only focus on a few conditions. Consequently, the integration of these small datasets is challenging and would introduce between-experiment noise. In comparison, this tilling array dataset is a unified dataset where experiments were conducted by different labs using standard operation procedures and experimental protocols. More importantly, all the probes were pre-processed together by adopting same approaches and quality control criteria which minimised the between-experiment noises.

**Figure 1:**
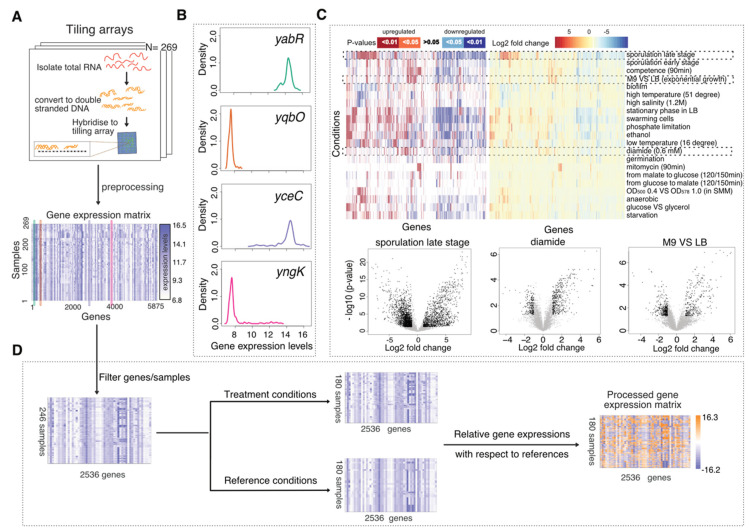
Tiling array data and data processing. (**A**) Tiling arrays which measured the RNA signals under various conditions were pre-processed to a gene expression matrix consisting of the gene expression levels of 5875 genes for 269 samples. (**B**) Different distribution patterns for gene expressions across samples. (**C**) *p*-Values and log2-fold-changes for the differential expression analysis performed on 21 pairs of contrasting growth conditions. Genes differentially expressed (*p*-Value < 0.05, log-fold-change > 1) for three condition contrasts—sporulation late stage against pre-sporulation stage (S0 vs. S3–S8), diamide treatment against non-oxidative environment (Oxctl vs. Diami), exponentially growing with glucose in M9 against in LB (LBGexp vs. M9exp)—are highlighted as black points, respectively. (**D**) Data processing steps include filtering some genes and samples, calculating the relative gene expression for treatment conditions with respect to their corresponding reference expressions. The processed gene expression matrix contains 180 samples and 2536 genes whose expression levels range from −16.2 to 16.3.

This tiling array dataset identified 5875 transcribed regions, including 4292 Genbank annotated coding sequences (AL009126.3) and 1583 new transcribed regions (named as S1 to S1583), where the underlying transcription signals for the probes within the transcribed regions are higher than threshold in at least one hybridisation. An aggregate expression index was computed for each of the transcribed regions as the median log2 expression signal intensity of probes lying entirely within the corresponding region. The log2 expression values were further pre-processed with quantile normalisation [49], rendering a gene expression matrix for machine learning data analysis (Figure 1A). Log transformation turns the data distribution from a highly right skewed distribution to a quasi-normal distribution. Quantile normalisation attempts to make the data distribution across samples identical, so as to reduce between-sample variations. The log2 expression values are deposited in Table 2 from http://genome.jouy.inra.fr/basysbio/bsubtranscriptome/, accessed on 1 March 2021. The raw data and pre-processed data can be downloaded in Gene Expression Omnibus (GSE27219).

### 2.2. Data Processing

Appropriate additional data processing was essential as our computational strategies involved unsupervised learning where noise or unwanted signals, rather than variations of biological interest, may be extracted. Firstly, we removed 137 genes that were considered invariant in all conditions, i.e., genes expressed at top 30% or bottom 30% of whole chromosome in all conditions, as they did not provide any information for distinguishing between transcriptional states. The expression distribution across samples for these invariant genes commonly showed the first two patterns in Figure 1B which are close to normal distribution with low dynamic range, while the expression distribution for the biomarker candidates usually showed the last two patterns in Figure 1B which are either very right skewed or left skewed.

To analyse the variance introduced by different sources we performed differential expression analysis on 51 paired groups of samples (Supplementary Table S2). These pairs of sample groups usually correspond to experimental treatment conditions versus the counterpart control conditions, but can also correspond to other varying factors, e.g., samples grown in LB medium versus M9 medium. For each pair, we ran moderated *t*-tests [50] to identify genes that differentially expressed in one group of samples against the contrasting group. We showed how different sets of genes were significantly upregulated or downregulated in response to different conditions (Figure 1C). Some treatments (e.g., sporulation and starvation) led to more differentially expressed (DE) genes and larger log2-fold changes than other treatments (e.g., mitomycin, diamide). In particular, genes involved in spore maturation during the late stages of sporulation were found to be active in other conditions too where they dominated the global transcriptional patterns, rendering the signals generated by some other conditions more difficult to detect in a fine-grained manner. Moreover, other factors such as cell density (e.g., OD_500_ 0.4 vs. OD_578_ 1.0), medium (e.g., M9 vs. LB) and growth phases that vary in different experiments may compound the data analysis. More details are provided in Supplementary Figure S1.

Guiding by the analysis results above, we further processed the data towards cancelling out these unwanted variances and enabling the detection of smaller variation introduced by conditions that resulted in fewer DE genes. Secondly, we removed 3225 DE genes and 23 samples related to late-stage sporulation which generated much larger variance than the other genes and samples so as to make variation caused by other conditions more obvious (resulting in the first matrix of Figure 1D). Upon this removal of features and data points, we saw the data points in the landscape were more spread out in Supplementary Figure S2A compared to Supplementary Figure S1A, providing a higher resolution of the data points that were originally stacked together. Thirdly, in order to remove the variation due to different growth medium, phases, density and other factors presented in different experiments we standardised the gene expression data to produce what we call “relative gene expression” profiles: We selected 180 samples that were applied with extra treatments or stress conditions as compared to the initial control conditions (Supplementary Table S1B–D) and calculated the relative gene expression matrix (last matrix in Figure 1D) as in Equation (1). *D*_(*i*,*j*)_ (Difference in expression signal intensities for gene i and sample j) represents the transcriptional signal changes at original scale (before log2 scaled) between the treatment sample and its corresponding reference sample, where *TE* denotes log2 Treatment gene Expression, *RE* denotes log2 Reference gene Expression and *NE* denotes log2 Normalised relative gene Expression matrix. The returned relative expression values are equivalent to log-fold-changes that represents the transcriptional ratio changes incurred upon the switch from control conditions to treatment conditions, where the absolute value of *D* is added with a small value to avoid -inf and then multiplied with the signs to enable positive values for upward changes and negative values for downward changes. The resulting landscape retained only treatment samples with aforementioned compounding factors largely relieved (Supplementary Figure S1C).

(1) $$D_{\left(i,j\right)}=2^{TE_{\left(i,j\right)}}-2^{RE_{\left(i,j\right)}}\;NE_{\left(i,j\right)}=Sign\left(D_{\left(i,j\right)}\right)*log_{2}\left[Abs\left(D_{\left(i,j\right)}\right)+1\right]$$

### 2.3. Dimension Reduction and Clustering

We use the Uniform Manifold Approximation and Projection (UMAP) algorithm [46] for dimension reduction to project our data in two-dimensions. This two-dimensional map describing sample patterns structured by their transcriptional profiles was regarded as the transcriptional landscape. From the UMAP representation of the data, we constructed a nearest-neighbour graph of the samples, and then applied the Leiden clustering algorithm [47], which detected communities within a graph, to identify clusters in the transcriptional landscape. Samples within a cluster are believed to present similar cellular states resulting from similar transcriptional profiles. This strategy to identify clusters from gene expression data is routinely used in other areas such as the analysis of single-cell RNA-seq data [51,52,53].

The choices of clustering algorithm, distance function, model parameters, and dimension reduction subspace in which clustering takes place can possibly lead to different solutions, but it is generally not possible to adjudicate unambiguously these between methods. Here, we tuned the model parameters from both dimension reduction algorithm UMAP and clustering algorithm Leiden to optimise the clustering solution. We first performed the grid search for a range of parameters (minimal distance- 0.08, 0.1, 0.15, number of neighbours- 10, 15, 20 in UMAP and resolution- 0.08, 0.1, 0.15, number of neighbours- 8, 10, 15 in Leiden) and validated resulted different clustering solutions by manually inspecting how much the solution was in line with prior known label and existing knowledge in the domain. The solution with highest number of clusters while not splitting biological replicates and samples sharing underlying molecular mechanisms was chosen as the final solution.

### 2.4. Feature Selection Method

We performed a feature selection process to identify the transcriptional biomarkers, i.e., a reduced set of genes that can pinpoint the cluster in the transcriptional landscape. This was achieved by iteratively removing specific features while optimising or maintaining the classification performance, where the features are the gene expression profiles and classes are labelled as previously learned clusters. In order to identify candidate genes with good discriminative power between clusters, we have used our own machine learning algorithm called RGIFE (Rank-Guided Iterative Feature Elimination). This algorithm was designed to identify reduced and highly discriminative panels of biomarkers from high-throughput omics data [48] and has shown good performance across a variety of scenarios including omics technologies (transcriptomics, proteomics) and diseases (cancer, osteoarthritis) [54,55]. RGIFE starts by considering all potential biomarkers as candidates, and iteratively some are dropped if their removal has no negative effect on the predictive capacity of the RandomForest (RF) machine learning algorithm. The predictive performance of the RF models is estimated from our data using stratified 8-fold cross-validation. This data partitioning process and predictive capacity estimation is performed within the RGIFE algorithm in order to select models having good predictive capacity on unseen data. The order in which features are removed is determined by the ranking of feature importance produced by RF as part of its training process. Initially RGIFE will attempt to remove blocks of 25% of the number of features. If the trial to remove a block fails (because its removal led to a model of worse quality) the algorithm will attempt to remove another block, following the ranking of features produced by RF. After five consecutive failed trials, the block size will be divided by 4 and the process will start again by attempting to remove the block at the bottom of the ranking. Once the block size is reduced to 1, the algorithm will stop after five unsuccessful trials and will return the remaining features as the final panel, hence automatically deciding when to stop the iterative feature elimination process. For a full description of the algorithm please see [48].

We validated the overall prediction power of this model by performing nested n-folds cross validation. The whole dataset was split into 8 folds (outer layer) to construct training datasets and test datasets while each training dataset was further split into 6 folds (inner layer) for model parameters tuning. The parameter configuration that achieved the best performance was chosen after scanning ‘maximum depth’ in 5, 8, ‘number of trees’ in 500, 1500 and 3000, ‘tolerate misclassification’ in 1, 2. Additionally, we performed stratified sampling to ensure each fold is balanced in classes and carefully chose the number of folds to ensure at least one sample from each class would be included in the test data for two layers of cross-validation. For extra robustness, we repeated this process 20 times. For each time, training datasets and test datasets were regenerated and the average performance of the model across the folds was recalculated. The performance was reported in terms of accuracy, recall, precision, f1 score and the roc curves for all classes.

The biomarkers were obtained by running the model on the full dataset after parameter hypertuning (‘maximum depth’ = 8, ‘number of trees’ = 1500, ‘tolerate misclassification’ = 1). As RGIFE and Random Forest classifier are both stochastic algorithms, we ran the model 100 times and selected an optimal solution which had: (i) best predicting performance in terms of f1 score, (ii) smallest panel size.

### 2.5. Statistical Tests: Differential and Enrichment Analysis

We performed differential expression analysis during both the data processing and cluster validation steps. The former step aims to study how genes may be differentially expressed under different conditions. We compared treatment conditions and control conditions carried out within an experiment which were the relevant biological factors we were interested in, as well as similar treatment conditions carried out in different experiments with other factors (such as growing medium and phase) varying. The latter step identifies genes which are more highly or lowly expressed in the samples of one cluster than in the remaining samples. Both analyses were carried out using Empirical Bayes moderated *t*-tests [50]. We selected the genes with Benjamini–Hochberg adjusted *p*-Value < 0.05, log-fold-change > 1 as the DE genes. We also performed Gene Ontology (GO) and KEGG pathway enrichment analyses using hypergeometric two-sided tests with Bonferroni correction in ClueGO [56]. The tests were based on GO_10.03.2015 and KEGG_11.03.2015 databases and only terms with *p*-Value < 0.05 were reported.

### 2.6. Knowledgebase: Gene Annotations, Transcriptional Regulatory Network, Gene Ontology, KEGG Pathways

To validate the biological significance of our cluster and biomarker results, we inspected the functions of genes differentially expressed in each cluster, together with their Gene Ontology and KEGG pathway. We integrated the regulatory network reconstructed by Jose et al. [57] and the Subtiwiki regulon database [58]. The curated regulatory network consists of 294 regulators and 2816 targeted genes, representing 30 different mechanisms of regulation such as transcriptional factors, RNA switches, riboswitches, and small regulatory RNAs. The network also includes 160 metabolites which are related to 1382 genes. Using this knowledge base, we studied how different regulators may have been playing roles in different cellular states represented by our identified clusters. We inferred the regulator activities in a cluster by looking at both the expression intensity level and expression percentage level of the genes regulated by corresponding regulators. The intensity level was computed as the average relative expression across the samples within a cluster. The percentage level was calculated as the proportion of samples sensitive to this regulator in a cluster—a regulator is activated (or repressed) in a sample if the average expression of the genes it regulates is higher (or lower) than the 70th (or 30th) percentile expression in the whole transcriptome.

### 2.7. Validation of the Biomarker Panel on Independent Datasets

To examine the effectiveness of our biomarker panel in classifying different cellular states induced by diverse conditions, we validated these biomarkers on 8 external gene expression datasets which studied 9 different treatment conditions against their corresponding control conditions (Supplementary Table S5). We trained classification models using our biomarkers as input features and performed Leave One Out cross-validation to assess if the panel of biomarkers can distinguish the treatment conditions from control conditions. Due to the small sample size (N = 7~21) of these datasets we used a simple classifier, linear Support Vector Machine (SVM), to lower overfitting risk. Given that the limited dataset sizes often produce overoptimistic estimation, we adopted additional steps for further evaluate the robustness of our biomarkers panel: (i) For datasets with class imbalance, we oversampled the minority class by applying Synthetic Minority Oversampling TEchnique (SMOTE) [59]. (ii) We performed stratified bootstrapping with replacement to regenerate the training dataset of same size and repeated the cross-validation evaluation 100 times with different bootstrap samples. (iii) We compared the prediction performance of our biomarkers with the prediction performance of 500 randomly selected gene panels of same size by running the Kolmogorov–Smirnov (KS) test, which assesses whether two empirical distributions are from same population.

## 3. Results

### 3.1. The Transcriptional Landscape Constructed by Condition-Dependent Transcriptomes Reveals Major Cellular States of B. subtilis

**Figure 2:**
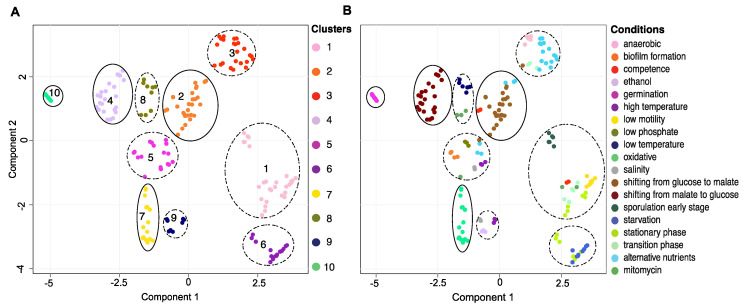
Transcriptional landscapes. (**A**) Transcriptional landscape in UMAP embedding, where samples (data points) with similar transcriptional profiles are positioned close to each other. Ten clusters were identified as indicated by colour. (**B**) Transcriptional landscape in UMAP embedding colour-coded according to the conditions in which they were grown. Clusters containing samples predominantly grown in similar conditions are circled with full lines. Clusters containing samples grown in multiple different conditions are circled with dotted lines.

To study the transcriptional changes in response to environmental conditions, we delineated the patterns of gene expression shifts underlining the condition-dependent transcriptomes in a landscape. This transcriptional landscape was constructed by transforming the high dimensional transcriptomics data into a two-dimensional map where samples undergoing similar transcriptional changes were positioned close to each other. We identified 10 clusters representing distinct changes in the transcriptional state, among which four clusters containing samples predominantly grown in similar conditions and six clusters containing samples grown in multiple different conditions (Figure 2, Supplementary Table S3A).

**Figure 3:**
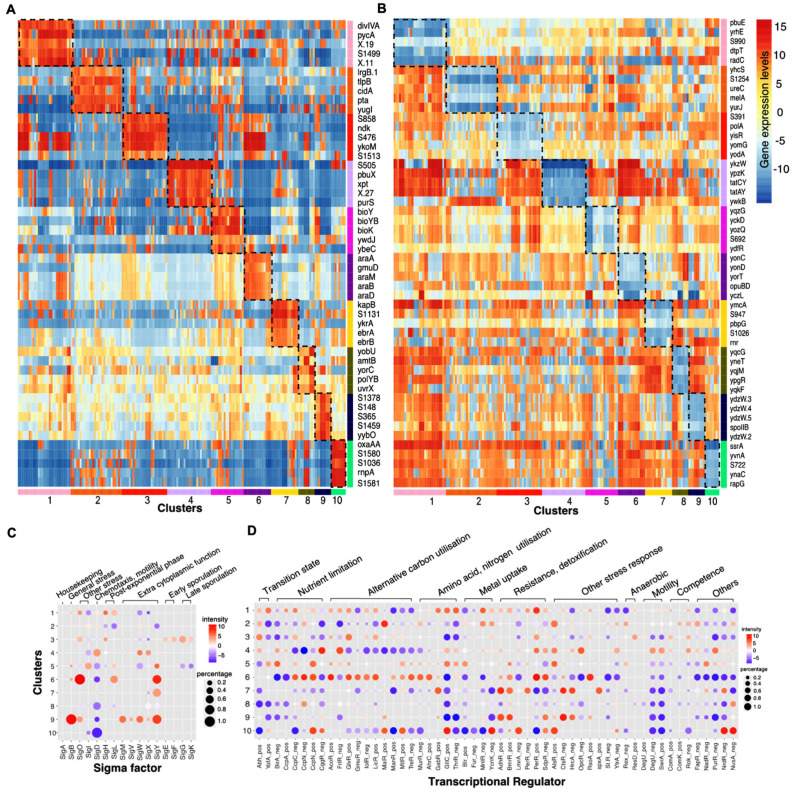
Cellular state inference for clusters. (**A**) Top5 (by *p*-Values) upregulated genes that are specific for each cluster. (**B**)) Top5 (by *p*-Values) downregulated genes that are specific for each cluster. (**C**) Inferred activities of selected sigma factors in each cluster. (**D**) Inferred activities of selected transcriptional regulators (TR) in each cluster. Dot colours represent the average intensity with red indicating activation while blue indicating repression. Dot sizes represent the percentage of samples within the cluster that sees the significant upregulated or downregulated expressions in genes targeted by that sigma factor or regulator. The sigma factor or regulator is activated (repressed) in a sample if the average expression of the genes regulated is higher (lower) than the 70th (30th) percentile expression in the whole transcriptome. The inferred activities for the full list of sigma factors and TRs are given in Supplementary Table S3E.

We further analysed the common genetic regulatory effects shared by each cluster coupling with the common physiological effects of the treatment conditions to infer the cellular growth states that the bacteria presented in different clusters. We studied the functions of genes that were significantly upregulated or downregulated in each cluster (Figure 3A, Supplementary Table S3B,C) and identified the corresponding enriched GO terms or KEGG pathways (Supplementary Table S3D). To obtain a global picture of how different sigma factors and transcriptional regulators (TR) played roles across clusters, we averaged the activities of genes regulated by each sigma factor or TR for each cluster, using both the percentage of samples that saw an increased or decreased expression in genes regulated by a given sigma factor or TR and the average intensity of gene expression (Figure 3B, Supplementary Table S3E). However, we note that this activity score of sigma factors or TR are based on the common increased or decreased expression in all the targeted genes, it is not sensitive to the sigma factor or TR that regulates large pool of genes, such as SigA, AbrB, SigE, etc.

Cluster 1 is associated with a range of conditions including 1 to 2 h after the induction of sporulation, 90 min after the induction of competence, transition to or in stationary phase (Lbtran, Lbstat, LBGtran, LBGstat), colonies or swarming cells growing on agar, and 45 min after treatment with the DNA damaging compound, mitomycin. In the majority of samples, increases were observed in the expression of genes activated by SigO, YofA and an over representation of the GO term cell communication, suggesting the bacteria were experiencing post-exponential growth and/or the induction of cellular differentiation. One common underlying factor in this cluster is the imposition of a starvation state, particularly of amino acids, as it sees a general increased biosynthesis of branched-chain amino acids [60]. The inclusion of the 45 min mitomycin treated samples may relate to the imposition of stress due to DNA damage [61] causing inhibition of DNA replication elongation and activation of the stringent response which are also seen in Amino acid starvation [62,63].

Cluster 6, containing samples grown to glucose starvation or stationary phase (M9stat, LPhT), is also associated with the imposition of a starvation state, although not only carbon starvation. As might be predicted, the full activation of SigO indicates the acidic environment that results, perhaps from overflow metabolites, in the stationary phase, while the repression of CggR (repressor of the glycolytic gapA operon) and BirA (repressor of biotin synthesis) indicates the nutrient limitation. The increased activities in SigL suggests the introduction of mechanisms to scavenge alternate nutrients through the synthesis of degradative enzymes such as levanase and enzymes for argenine catabolism. The increased expression of genes regulated by AcoR, LicR, TreR, AhrC also indicates the utilisation of overflow metabolites and other secondary carbon sources and amino acids including acetoin, lichenan, trehalose and arginine.

Cluster 3 represents conditions where the carbon source is changed from glucose to other compounds (glycerol, malic acid, pyruvate, gluconate, fructose) and anaerobic growing conditions, which both result in a radical shift in the metabolism. This cluster sees the upregulated expression in genes involved in respiration (*ctaC*/*ctaD*/*ctaE*/*ctaF*/*ctaG*/*qcrA*/*qcrB*/*qcrC*/*cccA*/*cccB*) as well as genes regulating aerobic and anaerobic respiration (*resD*/*resE*), and downregulated expression in genes involved in glutamate, threonine, purine synthesis (CltC switched off, ThrR and PurR switched on). 

Cluster 2 includes conditions in which malate was added to cells grown in glucose (GM), or cells induced for competence for 30 min (C30). Cluster 4, conversely, includes the conditions in which glucose was added to cells grown in malate (MG). Cluster 4 sees the reduced expression in the genes mediated by CcpA, CcpN which are related to carbon catabolite repression. This is because the genes necessary for the utilisation of less energetic carbon sources are repressed in the presence of glucose. Cluster 2 sees the opposite trend. In the conditions GM and M+G, cells relieve catabolite repression on the utilisation of malate as a carbon source as MalR accounting for malate uptake activates. The reason for the inclusion of C30 in this cluster is not immediately obvious but may be related to commonalities in the regulation of aromatic amino acid biosynthesis genes (*aroA*/*aroB*) during catabolite repression and early ComK mediated competence.

Cluster 5 contains samples exposed to high temperature at 51 °C (HiTm), high osmolarity (1.2 M sodium chloride, HiOs), low-phosphate, alternative carbon sources (fructose, gluconate, glutamic acid + succinic acid) and non-shaking treatment to form biofilms. Cluster 7 contains samples treated with compounds including diamide, paraquat and peroxide that induced oxidative stress. Cluster 9 contains samples treated with ethanol, high temperature at 48 °C (Heat) and 0.4 M sodium chloride (Salt). All three clusters include conditions that were likely to lead to the induction of the general stress response. This is the case especially for cluster 9 which sees the activation of SigB, SigY, SigM. However, the genes mediated by SigB are not generally upregulated for cluster 5 and cluster 7 as they were exposed to conditions in which additional physiological modifications were imposed. For cluster 7, apart from the activation of AdhR for detoxification and BmrR for multidrug resistance which were commonly observed in cluster 9, it additionally switched on CtsR, HrcA, PerP for oxidative stress response. Cluster 5 and cluster 9 both include heat or high salinity conditions. As expected, genes involved in DNA repair and heat, cold, osmotic or ethanol stress responses were increasingly expressed for both clusters, however, the number of regulated genes and upregulation levels were much lower for cluster 5 which contains samples treated with higher temperature or higher sodium chloride concentrations. This difference may be explained by the increased denaturation of proteins at the higher temperature of 51 degree, leading to an adaptive response to remove these denatured proteins in addition to the normal heat stress response. For example, differential expression of genes encoding resistance to osmotic down-shock (*yhdY*) and DNA repair (*mutT*) both indicate the presence of cellular damage. This cellular damage could be caused by the sample treatment at 51 degree or in 1.2 M sodium chloride medium.

Cluster 8 contains samples treated with low temperatures at 16 or 18 °C and 90 min of mitomycin. Common upregulated genes between these samples are involved in DNA repair (*uvr*/*ruvA*/*dinB*). This is because mitomycin is a potent cross linker of DNA which induces the DNA damage response [64] and evidence [65,66] also shows that nucleotide restructuring occurs in cold shock resulting in negative supercoiling of DNA, which also requires DNA excision repair. In addition, cluster 8 sees reduced expression in genes involved in synthesis of alternative amino acids or vitamins (*asnH*/*cysE*/*folEB*/*metA*/*pks*/*rib*) and cell division (*ftsW*/*glpG*), suggesting the slow growth which generally leads to reduced protein and DNA replication synthesis.

Cluster 10 contains samples taken during spore germination whose gene expression patterns are very distinct from normal vegetative growth in *Bacillus*. As expected, this cluster sees many enriched GO terms related to cellular metabolism. Compared to exponentially growth in LB, it also shows less expression in genes involved in TCA cycle (CcpC), mannose uptake (ManR) and more expression in genes involved in thymidine nucleotides/folate synthesis (CcpN), cell wall synthesis (*tagA/tagB*), glutamate biosynthesis (GltC).

### 3.2. A Small Subset of Genes Can Be Used as the Biomarker Panel to Indicate the Cellular State of B. Subtilis

**Figure 4:**
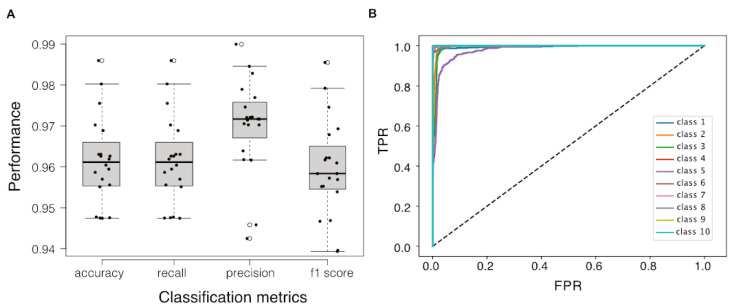
Class prediction performance with reduced features selected by RGIFE+RF model. (**A**) Boxplot of model performances in terms of accuracy, recall, precision and f1 score. (**B**) Receiver operating characteristic curves that reveal the relationship between true positive rate and false positive rate at various thresholds for predicting 10 classes, respectively.

As clusters of samples representing different cellular transcriptomic responses and growth states were revealed in the transcriptional landscape, we treated the identification of cellular state biomarkers as a process of selecting a minimised set of genes whose transcriptional profiles are able to distinguish between these clusters. We applied the RGIFE heuristic approach embedded with Random Forest Classifier (RGIFE+RF) to iteratively eliminate blocks of genes until the prediction power of the remaining genes degrades to an intolerable degree. We ran 20 repeats of nested n-folds validations to examine the overall performance of this RGIFE+RF model (i.e., the prediction power of the RF classifier built with only the genes selected by RGIFE) in which that the accuracy, recall, precision, f1 scores which were all higher than 0.94 (Figure 4A). The receiver operating characteristic curves of the classifier showed a high level of diagnostic ability for all classes except for class 5, where the area under the curve was slightly lower (Figure 4B).

Due to the stochastic nature of the RGIFE+RF model and the high level of correlation between many of the gene features, the set of genes selected by the model and the performance of the model may vary with each run. We ran the RGIFE+RF model one hundred times and discovered many alternative solutions that achieved comparable prediction performance with less than 20 genes, however, some genes were more frequently selected than others (Supplementary Table S4). In Table 1, we show the biomarker panel that achieved the best performance (n-folds cross validation within RGIFR showed f1-scores at 1.0) with smallest gene set size (10 genes).

**Figure 5:**
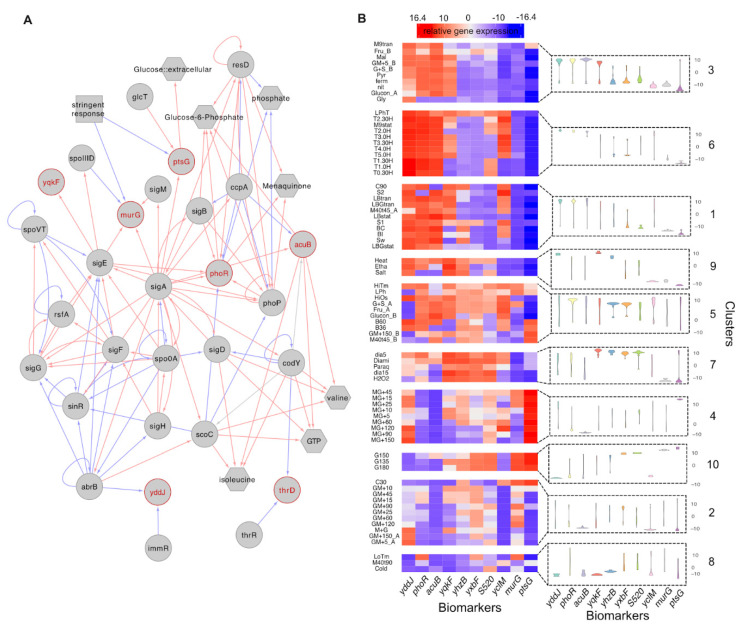
(**A**) The sub-regulatory network that connects the biomarkers (highlighted as red). Only 7 biomarkers were included in the complete *B. subtilis* regulatory network which consists of 294 regulators targeting 2816 genes and 160 metabolites related to 1382 genes. Red lines indicate activation while blue lines indicate repression. (**B**) The heatmap shows the average relative gene expression levels for identified biomarkers (columns) under different conditions (rows) which are grouped by clusters. As some biological replicates grown under the same conditions (e.g., M40T45) presented distinct transcriptional states and thus were grouped into different clusters, we re-labelled those conditions accordingly (e.g., ‘M40T45_A’ and ‘M40T45_B’). The violin plots present the patterns of biomarker expression distributions of samples included in corresponding clusters.

As might be expected, this biomarker panel includes members regulated by a large range of sigma factors and cellular regulators. The smallest connected regulatory network that connects seven of the biomarkers together demonstrates that these genes are distributed across the regulatory and metabolic networks of *B. subtilis* (Figure 5A). The rest of the biomarkers, including an antisense RNA, a putative transcriptional regulator and a coding sequence for conserved hypothetical protein, are less well studied but may have as yet to be identified as having important cellular functions. We visualised the variation in relative gene expressions for each of the biomarkers across clusters and within clusters (Figure 5B). It shows the potential utility of theses biomarker individually and as a set. In Supplementary Figure S2, we show in more detail how the individual biomarker profiles across each sample of the data. However, the random forest model used to identify biomarkers is a multivariate model, and the distinguishability of all clusters is based on the collective efforts of all biomarkers.

To validate if the biomarker panel remains effective at distinguishing different cellular states studied in independent studies, we tested their classification power to identify the transcriptomics profiles underlying nine environmental conditions from external datasets. Most of these conditions are similar to the ones (e.g., salinity stress, cold shock, heat stroke, oxidative stress) that were presented in the original dataset, while others are new conditions (such as pressure stress and deep starvation). Our biomarkers showed good performance at discriminating between treatment and control samples (f1-score > 0.9) for most of the conditions as shown in Table 2. Surprisingly, we also achieved good classification performance for new conditions, i.e., pressure stress and deep starvation, which were not seen in the training datasets used to identify our biomarkers. The exception was the antibiotic condition to which our biomarkers were less sensitive to.

In addition, we compared the distribution of biomarker performances across 100 repetitions of bootstrap resampling with the distribution of performances of 500 randomly chosen gene panels and calculated the Kolmogorov–Smirnov (KS) statistical test which indicates if the former can possibly be sampled from the later (Supplementary Figure S4A). In all datasets our biomarkers panel showed superior performance to the random panels, and all performance differences were significant according to the KS test. Although the genes in the biomarker panel work collectively to make predictions, their contributions varied across datasets. For example, gene *ptsG* played crucial role in distinguishing samples in heat stroke but was almost discarded for identifying pressure stress, and only five genes (*yqkF*, *thrD*, *murG*, *yddJ*, *ptsG*) were important for predicting cold shock samples (Supplementary Figure S4B).

## 4. Discussion

Bacteria experience cellular stresses when encountering unfavourable environments, nutrient limitation or protein production stress in laboratory scale-up experiments and industrial bioreactors. Monitoring the growth states and understanding the molecular activities within the cell population sheds lights on ways to relieve cellular stress and optimise product yield, however, current studies have been mostly confined in unravelling the signalling systems of individual treatment conditions and their corresponding gene regulatory systems. Making the most effective use of high throughput transcriptomics data collected under a wide range of conditions, our research deploys data-driven approaches to enhance the global understanding of transcriptional shifts in response to different conditions and to quantitatively extract a few key genes as the transcriptional biomarkers for sensing diverse cellular growth states presented in this transcriptomics data.

We achieved these goals with a set of data mining techniques where unsupervised learning was first performed to extract data representation-the clusters in the transcriptional landscape—and supervised learning-selection of features relevant to cellular growth states-was then performed with the ‘ground truth’ class labels set as the previously learned clusters. Caution was taken to prevent the clustering solution being driven by the nuisance variation and irrelevant biology. We explored the existing batch effects correcting and factor analysis methods including Combat [67], sva [68], sc-batch [69], mnnCorrect [70], but all failed to remove the unwanted variances introduced by different experiments, growing medium, or other unmodelled factors in the growth conditions. This is because the experiment design in our data does not meet the assumption of these algorithms, which requires that the factor of interest is not severely confounded with the other factors. Instead, our approaches are fairly simple in design but were successful in cancelling out most of the nuisance variation and irrelevant biology: We first removed the variances correlated to sporulation which are not a focus in this study and then standardize the gene expression values in treatment samples with respect to the reference gene expression values in their corresponding control samples.

Although there is no clear measure of success for the unsupervised learning methods, we validated the cluster solution using the existing knowledge, such as the gene regulatory network, gene annotation, KEGG and GO databases, to infer the underlying cellular growth states. The computational strategies designed in this study indicated the possibility of combining supervised learning methods with unsupervised learning processes, utilising domain knowledge to guide data-driven strategies or to assess the results.

The RGIFE model, which was used to identify the biomarkers, uses heuristics to find approximate solutions that do not guarantee convergence to a global optimum. We therefore run the model multiple times to find the best solution as well as several alternative solutions. Through the validation on external datasets, we showed how our biomarker panel was able to separate normal from treatment (i.e., stressed) cells under a broad range of stress conditions, among which pressure stress and deep starvation were beyond the conditions which were studied in the original dataset from which the panel was derived. This shows the potential of our biomarkers in identifying unknown, complicated stress conditions. However, we may still fail in distinguishing the conditions where only a few genes or unique genes were differentially expressed, e.g., our biomarkers showed worse prediction for the antibiotic condition. Nevertheless, we believe that analysing the expression of these biomarkers should allow most of the cellular state of the organism to be inferred in practice. For example, a biotech company can conduct rapid RNA-seq experiments to monitor the cellular states of a bioreactor or growing population. In the future, strains could be engineered with reporter genes linked to these biomarker genes for the in vivo real-time measurement of the bacterial cell transcriptional state. As the research is conducted in a data-driven manner, we would still expect laboratory experiments to solidly verify the biomarkers we identified.

Some limitations in this study and directions to extend the work are: (i) Validation of the biomarkers on a comparable independent dataset is missing. Although the tiling array dataset we used contains a broad range of conditions and therefore is ideal for exploring transcriptional landscape, it adopts old transcriptomics measuring technology, while most of the recently generated transcriptomic datasets are based on RNA-seq technology. Therefore, validating our biomarkers with a comparably large and diverse RNA-seq dataset is preferred. However, such dataset is difficult to find. The computational strategies we have designed for this work are not restricted to a specific dataset type and should be applicable to any new dataset generated with enough experimental conditions to infer a diverse expression landscape from it. A large and diverse dataset would allow our machine learning models to achieve good performance and enable the discovery of biologically meaningful information. (ii) This landscape is limited by the experimental design in the transcriptomics data we used. Additional samples grown, for example, under conditions that mimic industrial fermentations would help validate the biomarkers in an industrial setting. (iii) The generation of these landscapes is limited by the characteristics of the expression data. For example, the approach is unable to distinguish cellular states if only a few genes are differentially expressed at a low level. This situation may be improved in the future with additional feature engineering. (iv) With only transcriptomic data we are unable to analyse post-transcriptional activities which provide additional information about the physiological state of bacteria. These computational strategies can be applied at multi-omics scale to enhance the understanding of cellular activities at molecular levels.

In conclusion, we introduce a set of data mining methods designed to construct a landscape from transcriptomics data that reveals limited clusters of different transcriptional patterns and to identify an enough small panel of biomarkers that characterise the transcriptional state denoted by clusters in the landscape. We show that different transcriptional states (i.e., clusters) we identified for *B. subtilis* can be linked to different cellular growth states by analysing their biological significances and thus the transcriptional biomarkers that are distinguishable of these clusters can be exploited to sense cellular growth state of the bacteria. We believe these computational strategies have the potential to be applied to a wide range of applications involving the design of biosensors from data in which clear ground truth labels are missing.

## Figures and Tables

**Table 1:** Biomarker list.

Biomarker	Function	Product
*yddJ*	prevention of redundant transfer of ICEBs1 into host cells	ICEBs1 exclusion factor, putative lipoprotein
*phoR*	regulation of phosphate metabolism	two-component sensor kinase
*acuB*	unknown	unknown
*yqkF*	unknown	NADPH-dependent 4-Hydroxy-2,3-trans-nonenal reductase
*yhzB*	unknown	conserved hypothetical protein
*yclM (thrD)*	unknown	aspartokinase III
*yxbF*	similar to transcriptional regulator (TetR family)	unknown
*S520*	The antisense RNA of ygxB	5’UTR of mobA
*murG*	peptidoglycan precursor biosynthesis	UDP-N-acetylglucosamine-N-acetylmuramyl-(pentapeptide)pyrophosphoryl-undecaprenol N-acetylglucosamine transferase
*ptsG*	glucose transport and phosphorylation, control of GlcT activity, phosphotransferase system (PTS) glucose-specific enzyme IICBA component	glucose permease, trigger enzyme

**Table 2:** Validation Performance.

Test Condition	Sample Size		Accuracy		Recall		Precision		F1-Score		KS *p*-Value
	Treatment	Control	Mean	std	Mean	std	Mean	std	Mean	std	
Salinity stress	6	6	0.98	0.03	0.99	0.03	0.98	0.04	0.98	0.03	1.84 × 10^−7^
Glycine betaine	6	6	0.96	0.05	0.98	0.05	0.95	0.07	0.96	0.05	6.39 × 10^−3^
Heat stroke	12	9	0.89	0.04	0.92	0.05	0.88	0.05	0.90	0.04	<1 × 10^−10^
Cold shock	5	4	0.90	0.04	0.82	0.07	1.00	0.00	0.90	0.04	2.42 × 10^−9^
Oxidative stress	5	5	0.92	0.07	0.86	0.01	0.99	0.01	0.91	0.09	<1 × 10^−10^
Pressure stress	11	5	0.99	0.01	0.99	0.02	1.00	0.00	0.99	0.01	<1 × 10^−10^
Stationary phase	9	9	1.00	0.00	1.00	0.00	1.00	0.00	1.00	0.00	4.64 × 10^−4^
Antibiotic	9	9	0.78	0.06	0.89	0.09	0.79	0.07	0.78	0.07	1.89 × 10^−9^
Deep starvation	3	4	0.89	0.12	0.99	0.03	0.84	0.16	0.90	0.10	<1 × 10^−10^

## Data Availability

Data used for analysis can be downloaded in http://genome.jouy.inra.fr/basysbio/bsubtranscriptome (accessed on 1 March 2021). The Gene Expression Omnibus session numbers of the data used for validation are provided in Supplementary Table S5. The codes for data analysis are available in https://github.com/neverbehym/transcriptional-biomarkers-subtilis (accessed on 1 March 2021).

## Supplementary Materials

The following are available online at https://www.mdpi.com/article/10.3390/s21072436/s1. Supplementary Figure S1. The UMAP embedding of transcriptomics data before data processing; Supplementary Figure S2. The UMAP embedding of transcriptomics data after data processing; Supplementary Figure S3. The spectrum of biomarker gene expressions in the landscape; Supplementary Figure S4. The performance and feature importance in external validation; Supplementary Table S1. Samples and conditions; Supplementary Table S2. Differential expressed genes on different conditions; Supplementary Table S3. Clusters; Supplementary Table S4. Biomarkers; Supplementary Table S5. Validation datasets; The interactive Figures can be found in https://neverbehym.github.io/TB_InteractiveFigures.html (accessed on 1 March 2021).
