# Supplementary material for: Computational Strategies for the Identification of a Transcriptional Biomarker Panel to Sense Cellular Growth States in Bacillus subtilis
Source: Sensors (Basel). 2021 Apr 1;21(7):2436. doi: 10.3390/s21072436 (PMC8036383; doi:10.3390/s21072436)
Supplement: Supplementary file 1 [file sensors-21-02436-s001.zip › Supplementary Figures.docx]

**Supplementary Figure S1.** **(A)** The UMAP embedding of transcriptomics data without further data processing, where the colour indicates the main conditions treated to grow cells and the shape indicates either these conditions were considered as control or treatment in the experimental design. As circled with lines of the same colour as the data points, late-sporulation-stage/starvation data points (treatment samples) are positioned far away from before-sporulation/before-glucose-exhausted date points (control samples), suggesting large transcriptional changes may have incurred. By contrast, as circled with grey lines with-mitomycin/with-paraquat data points (treatment samples) and without-mitomycin/without-oxidative data points (control samples) stick together – subtle signals created by these condition changes were globally invisible. **(B)** The UMAP embedding of original transcriptomics data with colour indicating different medium in which cells were grown. It is notably that two divided groups of data points in the map are corresponding to LB medium and the other medium. **(C)** The UMAP embedding of original transcriptomics data with colour indicating different batches of experiments conducted. The patterns are organised based on experiments rather than conditions in some regions, e.g. for Experiment.13 and Experiment.15.

**Supplementary Figure S2.** **(A)** The UMAP embedding of reduced transcriptomics data, with invariant genes and the late-sporulation-related genes as well as samples were removed. The colour indicates the main conditions treated to grow cells and the shape indicates either these conditions were considered as control or treatment in the experimental design. Compared to the UMAP embedding of original transcriptomics data in Supplementary Figure1A, the data points further spread out and provide a better resolution. **(B)** The UMAP embedding of processed transcriptomics data after further standardisation completed. Only treatment samples were retained. **(C)** The UMAP embedding of processed transcriptomics data with colour indicating different medium in which cells were grown. The patterns are not dominantly driven by medium effects. **(D)** The UMAP embedding of processed transcriptomics data with colour indicating different batches of experiments conducted. The landscape is able to capture the biology underlying different conditions treated in Experiment.13 and Experiment.15.

**Supplementary Figure S3.** **(A)** The spectrum of biomarker gene expressions in the transcriptional landscape. The gene expression profiles of 10 biomarkers are portrayed with a colour scheme in the same UMAP embedding, the transcriptional landscape constructed with global gene expression shifts under various conditions. Red indicates upward transcriptional changes while blue indicates downward transcriptional changes with respect to conditional changes. **(B)** Cluster identity of data points is indicated by 10 colours in the same UMAP embedding of transcriptional landscape.

**Supplementary Figure S4.** **(A)** The density plots show the distributions of performances (F1-socre) for identifying samples under 9 different conditions across repetitions of cross-validation. The performances of biomarkers were obtained after 100 repetitions, and compared with the performances of 500 different sets of random genes from 500 tests. Kolmogorov–Smirnov (KS) test were performed to suggest how likely these two empirical distributions are from the same population. **(B)** The bar charts show the weights of all the features in each of SVM classifier used, indicating the feature (gene) importance to identify samples under 9 different conditions. The higher expression of genes coloured in blue give a positive sign while red gives a negative sign.
